# Magnification of the therapeutic uses of pomegranate fruits and peel in rats injected with carbon tetrachloride (Ccl4)

**DOI:** 10.1016/j.sjbs.2022.103374

**Published:** 2022-07-28

**Authors:** Lobna Saad Mohammed Abd Elmeged, Magbolah Salem Helal Alzahrani

**Affiliations:** a Department of Home Economics-Nutrition, AL-Baha University, AlMakhwa, Saudi Arabia; b Biology Department, Faculty of Science, AL-Baha University, Saudi Arabia

**Keywords:** Pomegranate fruits and peel, Nutritional value, Carbon tetrachloride, Hepatocellular, (POF), pomegranate fruits, (POP), pomegranate peel

## Abstract

The liver is more prone to infections that cause fibrosis, such as steatosis, non-alcoholic steatohepatitis, hepatotoxicity, cirrhosis, and hepatocellular carcinoma. Also, Viral hepatitis is a common condition worldwide it worsens into chronic inflammation of the liver. One of the healthiest fruits is the pomegranate for the body and health, as it contains a high nutritional value of minerals, vitamins, antioxidants, so we worked on this investigation to magnify the therapeutic applications of pomegranate fruits (POF) and peel (POP) in carbon tetrachloride-injected rats (Ccl4). The experiment was carried out in a caged animal. All rats were fed a basal diet for one week before the study, and they were divided into seven groups, each with six rats. As a control negative group (C–ve), the first group sample was fed only the basal diet for 28 days. The remaining rats (n = 36) were injected with carbon tetrachloride (Ccl4). Five groups were fed varying concentrations of (5 %, 10 %, 15 % POF, 5 %, and 10 % pomegranate peel (POP)), whereas one group was diagnosed with the illness and disease, and didn't even feed the experimental diet. The results revealed significant increases in T.BIL, D.BIL, and BIL in the serum of rats injected by CC14 to induce hepatopathy compared to the healthy group (normal rats). Also, the best treatment considering the serum D.BIL was recorded for the 5 % POF.

## 1 Introduction

Viral hepatitis is a common condition worldwide it worsens into chronic inflammation of the liver. The pomegranate, one of the most beneficial fruits for the body and health, contains minerals, vitamins, and antioxidants, and its numerous phytochemicals, including polyphenolic compounds such as hydrolyzable tannins, anthocyanins such as gallotannins and ellagitannins and gallotannins, and condensed tannins such as proanthocyanidins, have been discovered in various parts of the fruit. Pomegranate fruit contains hundreds of seeds surrounded by red liquid grains called arils, from which pomegranate juice can be prepared or added to the diet in a variety of ways to reap its benefits. Antioxidants are used to treat some diseases such as liver disease and remove toxins from the liver, (Soliman and Selim, 2012). Additionally, pomegranate leaves are anti-inflammatory and hepatoprotective (Lansky and Newman, 2007).

Pomegranate has been demonstrated to lower oxidative stress factors, which is likely due to its antioxidant activity, which is due to the free radical scavenging abilities of its phenolic components 3.17. In recent years, Pomegranate peels are considered a promising natural source of dietary fiber because it contains many phenols that show antioxidant activity, in addition to giving an acceptable color and smell, making it more suitable for use in a wide range of food fortification and obtaining healthy baked goods of high nutritional value (Ghazaleh et al., 2013). The researchers are focusing on liver fibrosis since the condition could be reversed in its early stages and the liver can be restored to its proper function, which is the fundamental goal of their research. Fibrosis of the liver is a dynamic pathological condition caused by an excess accumulation of extracellular matrix as a result of acute inflammation. This inflammation stimulates the formation of granulation tissue, but it also starts the wound-healing phase, which reduces inflammatory tissue destruction (CCI4), which is widely utilized to generate liver injury in rodents in experiments. In the centrical region, a single dose of CC14 produces steatosis and necrosis (Friedman et al., 2007).

In the centrical region, a single dose of CC14 produces steatosis and necrosis (Friedman et al., 2007). The creation of a metabolite, CCI4, which would be a major hepatotoxin responsible for alteration in cell permeability, causes liver toxicity during CC14 poisoning. It affects mitochondrial function, ultimately leading to cell death. Additionally, chronic CC14 exposure resulted in cirrhosis in rats *(*Chieli and Malvaldi, 1985*).* Several plants have been proposed to alleviate or care for liver problems, including Pumpkin seeds (Morsi, 1992).

Aim of study: Magnification of the therapeutic and immunological uses of pomegranate fruits and leaves in rats injected with carbon tetrachloride (Ccl4).

## 2 Materials and methods

### 2.1 Materials

#### 2.1.1 Preparation of POF and POP

Pomegranate fruits and peel are properly cleaned, cut into small slices, and dried for 3-days at 50 °C in a drying oven, before crushing and grinding into a powder form.

#### 2.1.2 Experiential animals

In this investigation, 42 male albino rats of the Sprague Dawley breed weighing 150 ± 10 g were used.

#### 2.1.3 Carbon tetrachloride (Ccl_4_)

El-Gomhoria Company for Chemical Industries in Cairo, Egypt, provided the compound (Ccl4) carbon tetrachloride in a 10 % liquid solution. It was distributed in white plastic water bottles holding one litre as a toxic substance ingredient for liver disease, according to (Passmore and Eastwood, 1986). It is diluted using paraffin oil obtained from the drugstore during the induction.

#### 2.1.4 Rats

mature male Sprague-Dawley albino rats, weighing (B.Wt)150–160 g. At the age of 14–16 weeks, the animals were transferred from the Animal Laboratory. The animals were kept in plastic cages with stainless steel covers and were kept in very clean conditions. For adaption, rats were given the basal diet for Seven days prior to the study. A smallmouth bottle connected with a metallic tube and a piece of plastic tubing at the mouth provided Ad libitum water. As previously indicated, rats were acclimatized on a 12-hour light/12-hour night condition for seven days prior to the beginning of the research to allow for acclimation.

### 2.2 Biological experiment

#### 2.2.1 Rats' normal diet

The basil diet contained 10 % casein, 0.25 % choline chloride, vitamin mixture (1 %), 5 % cellulose, 10 % maize oil, 4 % salt mixture, 0.35 % methionine, and corn starch (69.5 %) (Morsi, 1992).

The basal diet in the test contained CaCO_3_ (600 mg), MgSO_4_·2H_2_O (20_4_ mg), K_2_HPO_4_ (645 mg), CaHPO_4_·2H_2_O(150 mg), Fe(C_6_H_5_O_7_) 26H_2_O (55 mg), ZnCl_2_ (0.5 mg), MnSO_4_·4H_2_O (10 mg), NaCl(334 mg), CuSO_4_·5H_2_O (0.06 mg) and K_l_ (1.6 mg), (Hegsted et al., 1941).

The basal diet in the test contained Vitamin A (200 Iu), Vitamin K (0.50 Iu), Vitamin E (10 Iu), Calcium panthothenic acid (0.40 mg), Thiamin (0.50 mg), Pyridoxine (1.00 mg), Vitamin D (100 Iu), Folic acid (0.02 mg), Niacin (4.00 mg), para-amino – benzoic acid (0.02 mg), Choline chloride (200 mg), Inositol (24 mg), Vitamin B12 (2.00 g) (Campbell, 1963).

#### 2.2.2 Diet experiment

Table 1 shows the experimental diet, which is made up of the basic diet with powdered plants supplemented at a 10 % rate.Table 2..

**Table 1:** The basic and experimental diets' compositions.

**Component (g)**	**Basal diet**	**5 % POF**	**10 % POF**	**15 % POF**	**5 % POP**	**10 % POP**
**Test ingredients**	–	5	10	15	5	10
**Casein**	20	20	20	20	20	20
**Corn oil**	4.7	4.7	4.7	4.7	4.7	4.7
**Mineral mix**	3.5	3.5	3.5	3.5	3.5	3.5
**Vitamin mix**	1	1	1	1	1	1
**Cellulose**	5	5	5	5	5	5
**Cholin chloride**	2	2	2	2	2	2
**Sucrose**	10	10	10	10	10	10
**Corn starch**	**Up to 100**	**Up to 100**	**Up to 100**	**Up to 100**	**Up to 100**	**Up to 100**

**Table 2:** Shows the (BWG g), (FER), and (FI g/d) for the negative control, positive control, and other different groups of hepatitis rats fed on POF and POP.

**Groups**	**Parameters**		
	**B. W. G. (g)**	**F. I. (g)**	**F. E. R.**
Control (-)	0.51 + 0.04193 ^a^	0.023 + 0.00253 ^a^	22.06 + 0.303 ^a^
Control (+)	0.11 ± 0.032 ^d^	0.010 + 0.001 ^d^	11.5 + 0.55 ^d^
5 % POF	0.23 + 0.049^c^	0.014 + 0.0025^b^	16.9 + 0.25d ^c^
10 % POF	0.20 + 0.025^c^	0.013 + 0.0036^b^	15.3 + 0.47^c^
15 % POF	0.30 ± 0.030^b^	0.015 + 0.0015^b^	20 + 0.4^b^
5 % POP	0.30 ± 0.025^b^	0.014 + 0.0035^b^	22 + 0.53 ^a^
10 %POP	0.31 ± 0.026^b^	0.016 + 0.003^b^	19.1 + 0.56^b^
LSD	0.059	0.004	0.787

#### 2.2.3 Induction of liver intoxication in rats

Jayasekhar et al. (1997) used intramuscular injections of (Ccl4) carbon tetrachloride into paraffin oil 50 % V/V (2 ml/kg B.W.T.) twice per week for two weeks to induce chronic liver injury in male albino rats. Following Ccl4 injection, blood samples were obtained via the *retro*-orbital technique to confirm the presence of liver injury and to test liver function.

#### 2.2.4 Animal groups and experimental design

• Each group of six rats was divided into seven groups. The following were the rat groups:
• G1: Normal rats fed a basic diet for 28 days without any treatment as the positive control (Control group).
• G2: Rats with liver toxicity were kept as control negative and fed a basal diet for 28 days without any treatment.
• G3: Rats with liver intoxication and fed on basal diet plus 5 % of POF.
• G4: Rats with liver intoxication and fed on basal diet plus 10 % of POF.
• G5: Rats with liver intoxication and fed on basal diet plus 15 % of POF.
• G6: Rats with liver intoxication and fed on a basal diet plus 5 % POP.
• G7: Rats with liver intoxication and fed on a basal diet plus 10 % POP.

#### 2.2.5 Biological evaluation:

Each day for the 28-day study, the amount of food consumed was noted, and body weight was recorded each week. The feeding efficiency ratio (F.E.R.), the body weight growth (B.W.G. %), and the organ weight were all calculated (Chapman et al., 1959).

#### 2.2.6 Blood sampling:

At the end of this study, blood samples were obtained following a 12-hour fast. The *retro*-orbital approach with highly specialized glass tubes was used to collect blood samples into a dry clean centrifuge tube and let them to coagulate for half an hour in a water bath (37 °C) at room temperature. Before testing for glucose, blood samples have been centrifuged for 10 min at 3000 rpm to extract the serum. The residue was carefully aspirated, put into clean, tight-fitting polypropylene tubes, and maintained frozen until analysis at (−20 °C).

The liver, kidney, heart, and spleen were extracted and washed in salt solution before being weighed and preserved in 10 % formalin, as specified by (Drury and Wallington, 1967).

#### 2.2.7 Biological evaluation

Feed efficiency ratio (FER), food consumption, body weight gain % (BWG%), and FER (feed efficiency ratio). Using the equation following (Chapman et al., 1959):

$$using\;\;the\;\;equation\;\;below\;\;BWG\%=\frac{\mathrm{Final}\;\;Weight-Initial\;\;Weight}{\mathrm{Initial}\;\;Weight}\times 100$$

$$\mathrm{FER}=\frac{\mathrm{Gainin}\;\;\mathrm{Body}\;\;\mathrm{Weight}\;\;(g/day)}{\mathrm{Food}\;\;\mathrm{Intake}\;(g/day)}$$

$$\mathrm{The}\;\;relative\;\;\;Weight\;\;of\;\;organs=\frac{\mathrm{Organ}s^{\prime }s\;\;weight}{\mathrm{Animal}\;\;body\;\;weight}\times 100$$

#### 2.2.8 Analytical biochemistry

##### 2.2.8.1 Measurement of liver enzyme activity

A- Measurement of aspartate aminotransferase (AST) activity:

The AST enzyme was determined using a spectrophotometer and particular kits (BioMerieux) in accordance with Reitman and Frankel (1957).

B- Measurement of serum alanine aminotransferase (ALT) activity:

The calorimetric technique described was used to test the activity of the ALT enzyme (Reitman and Frankel, 1957).

C-Evaluation of serum alkaline phosphatase activity (ALP):

The colorimetric measurement of alkaline phosphatase (ALP) was performed according to Roy's method (1970).

D- Determination of serum total bilirubin:

Using a spectrophotometer set to 578 nm, total bilirubin in serum was quantified calorimetrically as described by Doumas et al. (1973).

E- Estimation of serum total cholesterol:

In accordance with Ratliff and Hall (1973), the total cholesterol level was evaluated.

F- Triglyceride measurement:

According to Jacobs and Van Denmark, the enzymatic colorimetric assessment of triglycerides has been established (1960).

G- Estimation of HDL:

Following Jacobs and Van Denmark's standards, HDL was tested (1960).

H- Determination of VLDL and LDL:

The following procedure was used to determine VLDL and LDL using Lee and Nieman's (1996) method.

##### 2.2.8.2 Statistical analysis.

A one-way categorization was used to calculate the statistical analysis. According to the least significant difference (LSD) and analysis of variance (ANOVA) (LSD) (Snedecor and Cochran, 1967).

## 3 Results

The main objective of this study to evaluate therapeutic value of POF and POP in rats administered carbon tetrachloride (Ccl4).

Biological changes: Effect of POF and POP on body weight gain (BWG)(g), the feed efficiency ratio (FER), and feed intake (FI)(g/d) of hepatopathy rats.

Body weight gain (BWG g) was 0.51 ± 0.041 (g) in the (C-ve) group, but 0.11 ± 0.032 (g) in the (C + ve) group; these data indicate a significant decrease in BWG (g) in rats intoxicated with CC14 (C + ve) group. When compared to the (C + ve) group, all rats infected by CC14 and fed on all tested POF and POP had a substantial rise in (BWG g). There were no significant differences in rats fed on (15 %, 5 % (POF), and 10 % POP), which were 0.30 ± 0.030, 0.30 ± 0.025, and 0.31 ± 0.026 (g), respectively; however, these mentioned components demonstrated significant increases when compared to another group. Furthermore, there were no significant differences in BWG (g) levels between rats fed on 5 % and 10 % POF, which were 0.23 ± 0.049 and 0.20 ± 0.025(g), respectively. The group with the highest BWG (g) was the 10 % POF group. This was similarly discovered for (FER), with the 5 % POP diet being the best group for (FI). In terms of EFR, data from the same table demonstrated that in rats injected with CC14 without diagnosis (C + ve) group (FER) was 0.010 ± 0.001, although in control rats it was 0.023 ± 0.0025. These findings indicate that there would be a significant decrease of (C + ve) in FER as compared with normal rats (C-ve). When compared to the (C + ve) group, all intoxicated rats fed POF and POP had a significant increase in FER. Data showed nonsignificant FER differences change between rats fed on 5 %, 10 m 15, POF and 10 % POF which were 0.014 ± 0.0025′ 0.013 ± 0.0036, 0.015 ± 0.0015, 0.014 ± 0.0035 and 0.016 ± 0.003, respectively, at the same time numerically, the 10 % POP group provides the highest increase as compared with the normal (+) group (0.010 ± 0.001). Finally, in the same table also feed intake FI results showed that the rats intoxicated with the CC14 (C + ve) group revealed 11.5 + 0.55 g/d when compared to the control rats (22.06 ± 0.30 g/d) group (C-ve). These results denoted that there was a significant decrease in the C + ve group. All intoxicated rats fed on POF and POP had a significant (p < 0.05) increase compared to the (C + ve) group. The best group was for rats fed on 5 % POP, which was 22.06 + 0.3O g/d; that’s the highest FI compared to other groups of pomegranates. Nicolle et al., (Nicolle et al., 2004) showed that liver disease could lead to malnutrition.

Poor dietary consumption, malabsorption and maldigestion, and alterations in the metabolism and retention of macro and micronutrients are the leading causes of malnutrition in people with liver disease. Elbanna, (Elbanna, 2014) found similar findings, stating that many cases of chronic or acute liver disease are ill and frequently lose weight.

It could be noticed that for normal rats (C-ve), the relative weight of liver, kidney, spleen, heart and pancreas were 2.5 ± 0.040, 0.6 ± 0.1, 0.3 ± 0.02, 0.48 ± 0.05 and 0.23 ± 0.04 (g/100 g B.Wt.), respectively, and in rats (C + ve), the relative weight of the previously referred to the organ was 5.1 ± 0.3, 0.95 ± 0.05, 0.79 ± 0.02, 0.93 ± 0.04 and 0.49 ± 0.03 (g/100 g B.Wt.), respectively. These findings showed that rats poisoned by CC14 had significantly higher relative liver, kidney, spleen, heart, and pancreatic weights than normal rats. For the relative weight of the liver, there was no significant (p < 0.05) difference between rats poisoned by CC14 and fed on 15 % on POF and 5 % on POF had, which were 2.6 + 0.45 and 2.4 + 0.45 (g/100 g), respectively. Rats given CC14 and fed on 10 % on POF have shown a higher nonsignificant increase in the mentioned relative organ weight than 5 % on POP and the control (+) group. The best group may be fed on a 15 % POF & 5 % POP diet, which is a nonsignificant difference between them. There were no significant (p greater than 0.05) differences in relative kidney weight between rats treated with CC14 and were fed on 5 %, 15 % POF and 5 % POP which was 0.7 ± 0.01, 0.69 ± 0.02 and 0.73 ± 0.04 (g/100 g), respectively. The best treatment was a 15 % POF diet, which revealed the lowest weight numerically. Concerning relative spleen weight, there was a nonsignificant change between rats injured by CC14 then fed on 5 % POF and 10 % POF which were 0.63 ± 0.04 0.63 + 0.04 (g/100 g) respectively. The best group of rats fed a % POP diet had the lowest potentially significant organ weight, that was 0.4 ± 0.02 g/100 g. According to data presented in the same Table 3, it is clear that relative heart weight showed nonsignificant change considering rats fed on 5 % POF and 10 % POF which were 0.79 ± 0.02 and 0.79 ± 0.03 (g/100 g), respectively. The best treatment seems to be found for the 15 % POF diet, which was numerically lowest compared to 5 % and 10 % POP groups. In the same Table 3, results can be seen that the relative pancreas weight showed a nonsignificant difference between 5 %, 10 % POF and 10 % POF groups which were 0.33 ± 0.04, 0.30 ± 0.01 and 0.30 ± 0.03 (g/100 g), respectively. The best treatment seems to be a 15 % POF diet, which revealed numerically less relative weight than 5 %, 10 % POP and control (-) groups. In this concern, contrary to the present results (Table 3), carbon tetrachloride CC14 lowered liver weight and promoted atrophy; relative weight is a parameter for the liver.

**Table 3:** Relative Organs Weight (g/100 g) for the negative control, positive control, and other groups of hepatitis rats fed on POF and POP.

**Groups**	**Parameters**				
	**Liver** **g/100 g**	**Kidney** **g/100 g**	**Spleen** **g/100 g**	**Heart** **g/100 g**	**Pancreas** **g/100 g**
Control (-)	2.5 ± 0.040^d^	0.6 ± 0.1^e^	0.3 ± 0.02^f^	0.48 ± 0.05^e^	0.23 ± 0.04 ^cd^
Control (+)	5.1 ± 0.3^ab^	0.95 ± 0.05^a^	0.79 ± 0.02^a^	0.93 ± 0.03^a^	0.49 ± 0.03^a^
5 % POF	4.5;0.5^ab^	0.7 ± 0.01^d^	0.63 ± 0.04^c^	0.79 ± 0.02^bc^	0.33 ± 0.04^b^
10 % POF	5.4 ± 0.45^a^	0.83 ± 0.04^b^	0.72 ± 0.02^b^	0.84 ± 0.04^b^	0.30 ± 0.0^bc^
15 % POF	2.6 ± 0.45^d^	0.69 ± 0.2^d^	0.48 ± 0.3^d^	0.68 ± 0.03^d^	0.20 ± 0.03^d^
5 % POP	2.4 ± 0.45^d^	0.73 ± 0.04 ^cd^	0.4 ± 0.02^e^	0.72 ± 0.03 ^cd^	02410.04 ^cd^
10 %POP	3.6 ± 0.47^c^	0.79 ± 0.03^be^	0.63 ± 0.04^c^	0.79 ± 0.03^bc^	0.30 ± 0.03^bc^
LSD	0.769	0.091	0.059	0.069	0.067

Values denote arithmetic + Standard deviation of the mean. Means with different letters (a,b,c,d,e,f,g) in the same column differ significantly at P < 0.05, while those with similar letters are non-significant by different.

### 3.1 Biochemical analysis

Effect of POF and POP on liver enzymes (ALT, AST, AST/ALT, and ALP) (U/L) of hepatopathy rats. Table 4 present the values of serum ALT, AST, ALP (U/L), and AST/ALT for the negative control, positive control, and other different groups of hepatitis rats fed on POF and POP.

**Table 4:** Effect of POF and POP on Liver Enzyme (AST, ALT, AST/ ALT, Alp) (U/L) of Hepatitis rats.

**Groups**	**Parameters**			
	**AST** **(U/L)**	**ALT** **IU/U**	**AST/ALT** **IU/U**	**ALP** **(U/U**
Control (-)	40.3 + 0.72*	18.1 + 0.36*	2 + 0.15^b^	219.1 + 0.66 ^g^
Control (+)	77.4 + 0.45^3^	29.1 + 0.45^3^	2.8 + 0.15^3^	394.3 + 0.43^a^
5 % POF	60.1 + 0.65°	23.2 + 0.32^c^	2.5 + 0.50^ab^	320.6 + 0.58^c^
10 % POF	63.2 + 0.62^b^	25.2 + 0.55^b^	2.5 + 0.55^ab^	324 + 0.45^b^
15 % POF	59.1 + 0.55^d^	22.3 + 0.49^d^	2.6 + 0.55^ab^	315 + 032 l^d^ ∼
5 % POP	52.1 + 0.37^6^	20.2 + 0.49^6^	2.8 + 0.20^ab^	290.2 + 0.62^6^
10 %POP	58.2 + 0.32^d^	23.1 + 0.56^c^	2.5 + 0.55^ab^	230 + 0.45*
LSD	0.959	0.825	0.741	0.906

Values denote arithmetic + Standard deviation of the mean. Means with different letters (a,b,c,d,e,f,g) in the same column differ significantly at P < 0.05, while those with similar letters are non-significant by different.

Data of Table 4 showed the highly significant increase in AST, ALT, ALT/AST and ALP from (40.3 ± 0.72 u/l, 18.1 ± 0.36 u/l, 2 ± 0.15, and 219 ± 0.66 U/L) respectively, in normal rats. (C-ve) group to (77.4 + 0.45 u/l, 29.1 + 0.45 u/l, 2.8 + 0.15 and 394.3 + 0.43(U/L) respectively, in hepatitis, inflicted rats, this change was due to the injection by CC14 had some adverse reactions and caused laboratory abnormalities in hepatopathy rats. In this concern, according to (Albakry, Lee, & Cham), increased aminotransferase (ALT and AST) activities indicate hepatocellular injury. Due to feeding on 5 %, 10 %, 15 % POF, 5% and 10 % POP noticeable decreases of AST, ALT. AST/ALT and ALP for rats were recorded compared with the control + ve group. Compared with the control (+) group, significant decreases for AST, ALT, ALT/AST, and ALP were recorded, particularly for the 5 % POF group. Table 5 present the level of total bilirubin, direct bilirubin, and indirect bilirubin(mg/dl) for the negative control, positive control, and other different groups of hepatitis rats fed on POF and POP.

**Table 5:** Effects of POF and POP on Hepatitis-induced rat bilirubin levels (total, direct, and indirect (mg/dl)).

**Groups**	**Parameters**		
	**T. Bil** **(mg/dl)**	**D.Bil** **(mg/dll**	**In. Bil** **(mg/dll**
Control (-)	5.6 + 0.55^b^	5.5 + 0.55^1^*	0.1 + 0.02°
Control (+)	7.5 ± 0.4^a^	7.2 + 0.45^3^	0.41 + 0.36^3^
5 % POF	6.6 + 0.45^ab^	6.3 + 0.47^ab^	0.28 + 0.32^b^
10 % POF	6.5 ± 0.55^ab^	6.2 + 0.47^ab^	0.27 + 0.049^b^
15 % POF	6.3 + 0.47^ab^	6.0 + 0.65^abc^	0.32 + 0.25^b^
5 % POP	5.7 ± 0.66^b^	5.4 + 0.30^c^	0.29 + 0.015^b^
10 %POP	6.3 ± 0.45^ab^	6.0 + 0.36^abc^	0.3 + 0.01^b^ •
LSD	0.902	0.840	0.051

Values denote arithmetic + Standard deviation of the mean. Means with different letters (a,b,c,d,e,f,g) in the same column differ significantly at P < 0.05, while those with similar letters are non-significant by different.

It's certain that the serum of T.BIL, D.BIL and In. BIL in (C + ve) group were 7.5 ± 0.4, 7.2 ± 0.45 and 0.41 ± 0.036 (mg/dl) respectively. Whereas in the control (-ve) group of control rats were (5.6 + 0.55, 5.5 + 0.55 and 0.1 + 0.02 mg/dl) respectively. These findings indicated that there were significant increases in T.BIL, D.BIL and In. BIL in the serum of rats injected by CC14 to induce hepatopathy compared to the healthy group (normal rats). In rats injected by CC14 and fed on 5 %, 10 %, 15 % POF, 5 % 10 % POP (7.5) %, there were significant decreases in serum level T.BIL, D.BIL., and In. Bil. For T.BIL rats fed on 5 %, 10 %, 15 % POF and 10 % 5 %, 10 %, 15 % POF showed nonsignificant difference between them but the best treatment considering the serum T.BIL was recorded for 5 % POF group (5.7 + 0.66 mg/dl). For D.BIL rats fed on 5 %, 10 % POF, and 10 % POF showed a nonsignificant difference between them, and nonsignificant differences determined between 15 % POF groups and 5 %, 10 % POP. The best treatment considering the serum D.BIL was recorded for the 5 % POP group (5.4 + 0.30 mg/dl).

Finally, rats fed on 5 %, 10 %, 15 % POF and 5 %, 10 % POP showed nonsignificant differences between them as regards In.BIL in serum of rats. The best treatment considering the serum In.BIL seemed to be the 10 % POP group (0.27 ± 0.049 mg/dl). Srivastava *et al*., (Srivastava et al., 2002) reported that plasma bilirubin is a measure of ability to transport bile, and the deposition of bilirubin in the tissues causes jaundice when the level is usually more than 2.5 mg/dl in serum.

Effect of POF and POP on HDL-C, LDL-C, VLDL-C (mg/dl), and Al of hepatopathy rats: The serum of lipoprotein components is shown in Table 6 (HDLc, LDLc, VLDLc, (mg/dl) and (Al ratio) for the negative control, positive control and other different groups of hepatitis rats fed on POF and POP.

**Table 6:** Effect of POF and POP on *HDL, VLDL, LDL (mg/dI), and* AI of Hepatitis rats.

	**Groups**			
**Parameters**	**VLDL** **(mg/dl)**	**HDL** **(mg/dl)**	**LDL** **(mg/dl)**	**AI** **ratio**
Control (-)	12.1 ± 0.26^e^	42.1 + 0.36^3^	23.9 + 0.32^8^	0.86 + 0.047°
Control (+)	19.1 + 0.36*	26.9 + 0.15*	113.9 + 0.15^a^	4.94 + 0.49^3^
5 % POF	17.2 + 0.32^b^	34.4 + 0.45^d^	70.4 + 0.36°	2.55 + 0.25°
10 % POF	16.1 + 0.49°	30.6 + 0.58°	88.9 + 0.36^b^	3.42 + 0.40^b^
15 % POF	15.6 + 0.5 l^d^	36 + 0.1°	34.4 + 0.45^d^	1.39 + 0.026^d^
5 % POP	15.0 + 0.55°	39.4 + 0.45^b^	29.9 + 0.26*	1.14 + 0.1^d^
10 %POP	16.6 + 0.32°^d^	34.9 + 0.15^d^	31.8 + 0.45°	1.39 ± 0.036^d^ ^
LSD	0.730	0.640	0.615	0.460

Values denote arithmetic + Standard deviation of the mean. Means with different letters (a,b,c,d,e,f,g) in the same column differ significantly at P < 0.05, while those with similar letters are non-significant by different.

It is clear that the significantly increased mean values for (C + ve) group in serum level for VLDLc, LDLc and Al, which were (19.1 + 0.36 mg/dl, 113.9 + 0.15 mg/dl and 4.94 + 0.49) being higher than respective means of control (-) rats. The contrast in serum level of HDLc was shown to be significantly (p < 0.05) decreased in the C + ve group, which was 26.9 + 0.15 mg/dl compared to the C-ve group, being 42.1 + 0.36 mg/dl.

Effects of POF and POP on hepatopathy rats' kidney function (Serum Urea, Serum Creatinine, and Serum Uric acid) (mg/dl): Table 7 reflected the serum concentration of creatinine (mg/dl), urea (mg/dl) and uric acid (mg/dl) for negative control and positive control and other different groups of hepatitis rats fed on POF and POP.

**Table 7:** Effect of POF and POP on Kidney Function (Serum Creatinine, Serum Urea, and Serum Uric Acid) (mg/dl) of Hepatitis rats.

Groups	Parameters		
	Creatinine mg/dl	Urea mg/dl	Uric Acid mg/dl
Control (-)	0.35 ± 0.035^b^	16.4 ± 0.55 ^g^	1.7 ± 0.5^b^
Control (+)	0.67 ± 0.02^a^	34.6 ± 0.55^a^	3.0 ± 0.15^a^
5 % POF	0.34 ± 0.04^b^	27.3 ± 0.7^c^	1.8 ± 0.3^b^
10 % POF	0.38 ± 0.03^b^	28.3 ± 0.47^b^	l.9 ± 0.4^b^
15 % POF	0.33 ± 0.04^b^	22.3 ± 0.50^e^	1.6 ± 0.55^b^
5 % POP	0.31 ± 0.05^c^	19.2 ± 0.61^f^	1.5 ± 0.35^b^
10 %POP	0.34 ± 0.02^b^	24.4 ± 0.45^d^	1.7 ± 0.47^b^
LSD	0.067	0.972	0.721

Values denote arithmetic + Standard deviation of the mean. Means with different letters (a,b,c,d,e,f,g) in the same column differ significantly at P < 0.05, while those with similar letters are non-significant by different.

Data of Table 7 showed significant increase in serum creatinine, serum urea and serum uric acid for (C + ve) group (inflicted rats with hepatitis) which were 0.67 + 0.02, 34.6 ± 0.55 and 3.0 ± 0.15 (mg/dl), respectively as compared with (C - ve) group (normal rats); value of control (-) group were 0.35 + 0.035, 16.4 ± 0.55 and 1.7 ± 0.51 (mg/dl), respectively. In the same Table 7, feeding on p POF and POP revealed nonsignificant differences between mentioned groups which fed on all groups of POF and POP and injected by CC14 for a concentration of S. creatinine and S. uric acid.

It should be noted from Table 7 that in the case of S. creatinine and S. uric acid selected best treatment reduced values of these compounds to fewer levels compared to (C-ve) group, showing a pronounced therapy and complete recovery towards the normal healthy rats, so the best group showing the least level compared to all groups of POF and POP for creatinine, urea, and uric acid was the 5 % POP group indicating values of 0.31 + 0.05′ 19.2 + 0.61 and 1.5 + 0.35(mg/dl), respectively. Bennett et al. (1995) showed that creatinine is dependent on the renal function capacity also showed that l-isothiocyanato-3-(methylsuphinly)-propane (IMSP or Iberian) is a significant component of glucosinolate hydrolysis present in cruciferous vegetables and POP. However, no effect was found on the liver or kidney function, when no changes in haematocrit, blood urea nitrogen, electrolytes, creatinine, or serum enzymatic activities have been shown to be physiologically significant. However, Table 8 presents the fasting serum sugar (glucose) (mg/dl) for the negative control, positive control, and other different groups of hepatitis rats fed on POF and POP.

**Table 8:** Effect of POF and POP on glucose (mg/dl) of hepatopathy rats.

Groups	Glucose. mg/dl
Control (-)	76.1 + 0.26^6^
Control (+)	158.2 + 0.58^3^
5 % POF	82.2 + 0.73°
10 % POF	86.2 ± 0.73^b^
15 % POF	80.1 + 0.75^d^
5 % POP	70.1 + 0.56*
10 %POP	79.2 + 0.83^d^
LSD	1.162

Values denote arithmetic + Standard deviation of the mean. Means with different letters (a, b, c, d, e, f, g) in the same column differ significantly at P < 0.05, while those with similar letters are non-significant by different.

From the above-mentioned data significant (p < 0.05) increase of control sample + ve group (158.2 ± 0.58 mg/dl) as compared to control-ve group (76.1 ± 0.26 mg/dl) was recorded. When rats were fed on POF and POP, the maximum glucose reduction recorded for 5 % POP group was 70.1 + 0.56 (mg/dl) compared to the C + ve group, it is clear that the 5 % POP group gave better results than other parts.

### 3.2 Discussion

The pomegranate, one of the most beneficial fruits for the body and health, contains minerals, vitamins, and antioxidants, and its numerous phytochemicals, including polyphenolic compounds such as hydrolyzable tannins, anthocyanins such as gallotannins and ellagitannins and gallotannins, and condensed tannins such as proanthocyanidins, have been discovered in various parts of the fruit. Pomegranate fruit contains hundreds of seeds surrounded by red liquid grains called arils, from which pomegranate juice can be prepared or added to the diet in a variety of ways to reap its benefits. Antioxidants are used to treat some diseases such as liver disease and remove toxins from the liver, (Soliman and Selim, 2012) phenolic compounds (such as in pomegranate and cabbage) have reduced the increase in serum levels of AST and ALT. Moreover, POF and POPs affect total bilirubin, direct bilirubin and indirect bilirubin (mg/dl) of hepatitis rats (Eberhart, 1986; Bennett et al., 1995), who discovered that pretreatment with cauliflower flavonoids increased the antioxidant activities of the liver and reduced bilirubin levels when compared to control groups. Glore et al. (1994) who showed pomegranate, oat bran, rice bran, legumes, broccoli, cabbage, carrots, cauliflower, and com are good sources of soluble fibres which bind excess cholesterol and carries it out of the body. Bennett et al. (1995) showed that creatinine is dependent on the renal function capacity also showed that l-isothiocyanato-3-(methylsuphinly)-propane (IMSP or Iberian) is a significant component of glucosinolate hydrolysis present in cruciferous vegetables and POP. However, no effect was found on the liver or kidney function, when no changes in hematocrit, blood urea nitrogen, electrolytes, creatinine, or serum enzymatic activities have been shown to be physiologically significant. Soliman and Selim (2012) showed that broccoli, pomegranate, and cabbage are the richest food sources of the trace metal chromium, which controls insulin and blood sugar. Pomegranate is mostly safe and does not lead to any side effect when taken as a fruit or juice. However, pomegranate extracts may lead to sensitivity in some cases. The symptoms of sensitivity include itching, swelling, runny nose, and difficulty breathing. There are some conditions when people need to be careful before taking pomegranate in any form.

Recommendations.

1. For hepatic patients, different POF and POP levels are recommended.
2. POF in different concentrations, especially 10 % POP, can treat diabetes.
3. Different POF concentrations can be recommended for atherogenic index levels and decreasing LDL.

## Declaration of Competing Interest

The authors declare that they have no known competing financial interests or personal relationships that could have appeared to influence the work reported in this paper.
